# Systematic review of differential methylation in rare ophthalmic diseases

**DOI:** 10.1136/bmjophth-2019-000342

**Published:** 2019-11-13

**Authors:** Katie Kerr, Helen McAneney, Laura Smyth, Cheryl Flanagan, Julie Silvestri, Micheal Andrew Nesbitt, Christopher Wooster, Amy Jayne McKnight

**Affiliations:** 1 Centre for Public Health, Institute of Clinical Sciences B, Royal Victoria Hospital, Queen's University Belfast School of Medicine, Dentistry and Biomedical Sciences, Belfast, UK; 2 The 100,000 Genomes Project Team, Belfast Health and Social Care Trust, Belfast, UK; 3 Department of Ophthalmology, Belfast Health and Social Care Trust, Belfast, UK; 4 School of Biomedical Sciences, Biomedical Sciences Research Institute, Ulster University, Belfast, UK

**Keywords:** genetics, retina, cornea, vision, public health, choroid, degeneration

## Abstract

Rare ophthalmic diseases have a devastating impact on a patient’s vision and consequently negatively affect their independence, ability to work and overall quality of life. Methylation is an important emerging biomarker of disease and may improve understanding of rare ophthalmic disorders. This systematic review sought to identify and evaluate literature on methylation and rare ophthalmic disease. MEDLINE, EMBASE, PubMed, Cochrane Database of Systematic Reviews and grey literature resources were searched for publications prior to 20 August 2019. Articles written in English which featured key terms such as ‘methylation’ and rare ophthalmic diseases were included. Titles, abstracts, keywords and full texts of publications were screened, as well as reference lists for reverse citations and Web of Science ‘cited reference search’ for forward citation searching. Study characteristics were extracted, and methodological rigour appraised using a standardised template. Fourteen articles were selected for full inclusion. Rare ophthalmic conditions include congenital fibrosis of extraocular muscles, retinitis pigmentosa, Fuchs endothelial corneal dystrophy, granular corneal dystrophy, choroideraemia, brittle cornea syndrome, retinopathy of prematurity, keratoconus and congenital cataracts. Outcomes include identification of methylation as contributor to disease and identification of potential novel therapeutic targets. The studies included were heterogeneous with no scope for meta-analysis following review; a narrative synthesis was undertaken. Differential methylation has been identified in a small number of rare ophthalmic diseases and few studies have been performed to date. Further multiomic research will improve understanding of rare eye diseases and hopefully lead to improved provision of diagnostic/prognostic biomarkers, and help identify novel therapeutic targets.

## Introduction

### Rare diseases are challenging

Rare diseases are defined by the European Commission as conditions which affect fewer than 1 in 2000 people, and in America as one which affects less than 200 000 people.^1^ Rare diseases affect 3.5 million people in the UK, 30 million people across Europe, 25–30 million people in the USA and 350 million people worldwide.^2^ To put this in perspective, 2.5 million people are living with cancer in the UK,^3^ and in 2015, a total of 101 200 people were diagnosed with HIV.^4^ Cumulatively, this is still less than the 1 in 17 people affected by a rare disease in the UK. While individual disorders are rare, collectively rare diseases pose a significant healthcare problem. These conditions are often life threatening and debilitating with ~30% of these patients with rare disease dying before they reach their fifth birthday.^5^ A significant issue for patients is in obtaining a diagnosis, with two in five individuals struggling to obtain an accurate and timely diagnosis, often encumbered by several misdiagnoses before the correct one is obtained.^6^ Even where a diagnosis is obtained, knowledge about a disease is often extremely limited by primary healthcare professionals, with treatment options even more so.

### Early diagnosis and intervention is crucial for ophthalmic diseases

For many ophthalmic conditions, early diagnosis is crucial to preventing further degeneration in patient eyesight, as well as for optimal management and support of the patient. Of the approximately 285 million cases of visual impairment worldwide, including both common and rare diseases, four out of five cases are thought to be preventable. The global initiative ‘VISION 2020: The Right To Sight’ seeks to reduce avoidable blindness at 25% by 2019 by collecting evidence on visual impairment, training of eye care professionals, provision of eye care and elimination of social and economic barriers to eye care.^7^

The lack of existing rare ophthalmic disease registries to provide a comprehensive and exact figure of the total number of rare eye disorders presents a challenge to researchers hoping to collate what evidence is available. However, a large number of conditions have been documented by several sources, including: The 100,000 Genomes Project ophthalmic inclusion criteria,^8^ the Genetic and Rare Diseases Information Center,^9^ the National Eye Institute,^10^ the Retina International and the Irish Target 5000—Gateway to Vision.^11^ These conditions range from rod/cone abnormalities, macular dystrophies, photoreceptors and bipolar cell abnormalities, vitreoretinopathies and hereditary choroidal diseases.^12^

Diabetic retinopathy, a leading case of vision loss and adult blindness worldwide, is an example of a common complex ophthalmic disease where early diagnosis and treatment reduces the risk of blindness. This is evident in the success eye screening programmes and increased glycaemic control have had in reducing adult blindness caused by diabetic retinopathy in England and Wales, with inherited retinal dystrophies now taking over as the leading cause of adult blindness.^13^ Furthermore, eye screening programmes in Iceland have reduced diabetic retinopathy blindness from 2.4% to 0.5% between 1980 and 1994.^14^ Therefore, the identification of biomarkers that can help develop early diagnostic tests, stratify patients and predict disease progression is urgently required for other ophthalmic diseases, especially rare diseases, which can cause vision loss and do not have existing screening programmes. One such progressive rare ophthalmic disease is Leber congenital amaurosis (LCA), which begins in childhood and vision deteriorates as the patient ages due to a lack of early diagnosis and treatment. Treatment options are limited for the majority of rare ophthalmic diseases, but recent gene therapy trials have shown promising results in restoring vision to nearly blind patients with LCA and in other inherited retinopathies, such as choroideraemia.^15^

### Next-generation multiomic analysis of rare disease

Multiomics is a biological approach to research which looks at multiple ‘omic’ terms, of which there are currently over 500.^16^ One ‘omic’ term, genomics, has been a key player in ophthalmic disease research; including the identification of disease-related genes and genomic areas in age-related macular degeneration, diabetic retinopathy and glaucoma.^17^ Crucially, whole-genome and whole-exome sequencing have enabled the prediction that as many as 3500 genes thought to cause rare disease (including rare ophthalmic disease) might be identified.^18^ Projects such as the UK 100,000 Genomes Project have been fundamental in introducing genomics into the healthcare system, with ongoing multiomics contributing to maximising diagnostic yield and improving our understanding of rare disease.^8^ This project aims to identify genomic variation such as a copy number variation and single nucleotide polymorphisms to provide diagnosis where patients have not received one already, improve our understanding of disease pathogenesis and identify novel treatment targets.

The National Ophthalmic Disease Genotyping and Phenotyping Network (eyeGENE) is an American/Canadian research body, created by the National Eye Institute, with the central aim of improving understanding of rare ophthalmic diseases, providing clinical and molecular diagnosis to patients and ultimately identification of therapeutic targets.^19^ As of December 2017, eyeGENE had an encouraging 130 publications, many of which were studies researching genetic mutations in rare ophthalmic disease.^20^ These publications include three review articles which highlight the promise for future retinal disease research using next-generation sequencing technologies; thus identifying a need to combine classical genetic mutation research with these high-throughput techniques to generate and analyse data on ophthalmic diseases.^21–23^ This fits with an expansion of research characterising several human diseases, including ophthalmic disease, at the molecular level using next-generation sequencing technology and a more informative integrated multiomic analysis.^12^

While genomic research has been the primary focus of ophthalmic research and indeed many other areas of human disease, several different types of ‘omic’ analyses are increasingly conducted in ophthalmic disease research. These include transcriptomics (the study of sum RNA levels),^24^ metabolomics (study of tissue metabolites),^25^ proteomics (protein analysis)^26^ and epigenomics (the study of variations in the DNA which are not a result of sequence-level modifications).^27^

### Methylation: a key epigenomic marker of human disease

Methylation, an important epigenomic biomarker, is a reversible chemical modification often seen at cytosine residue in CpG dinucleotide sequences in DNA. Specifically, it occurs when DNA methyltransferases transfer a methyl group to the fifth carbon position on a cytosine from S-adenyl methionine, forming 5-methylcytosine.^28^ Changes in DNA methylation may result from somatic or environmental factors as well as heritable factors and these changes can persist both in the long or short term, with the potential for transgenerational inheritance. It is associated with repressive effects on gene activity; however, the function of DNA methylation has been shown to vary depending on the genomic situation.^29^ Methylation has been investigated as a marker of many diseases including several cancers,^30^ metabolic disorders such as hyperglycaemia,^31^ neurological conditions such as autism spectrum disorder^32^ and, importantly, several common eye conditions such as diabetic retinopathy,^33^ age-related macular degeneration^34^ and cataracts.^35^

Therefore, in addition to the classical genetic and genomic research described above, there is a need to assess the potential for methylation as an epigenomic marker of rare ophthalmic disease. If methylation was found to be an accurate and sensitive epigenomic marker, it has the potential to contribute to early diagnosis, management and reduction of vision loss; as well as identify novel therapeutic targets to improve treatment options for patients with rare ophthalmic diseases.

### Aim and objectives

This review aimed to determine what evidence currently exists of differential methylation in rare eye diseases by:

- Systematically uncovering what, if any, current literature exists relevant to methylation and rare ocular diseases.
- Determining the method of methylation measurement and methodological rigour of any such studies.
- Highlighting any existing discussion of applying differential methylation as a biomarker of rare eye disease.

## Methods

This review was conducted using the Preferred Reporting Items for Systematic Reviews and Meta-Analyses Transparent Reporting of Systematic Reviews and Meta-Analyses Checklist,^36^ following our registered protocol, PROSPERO ID: CRD42018094231.^37^

### Eligibility criteria

Journal articles published before 20 August 2019 were included if they were quantitative, written in English and discussed both differential methylation and rare eye diseases. This is an updated search from the original date specified in the registered protocol, though all search terms and eligibility criteria remained the same.^37^ The focus of this review was non-tumorous eye diseases; therefore retinoblastoma, uveal melanoma and intraocular melanoma were excluded. Conditions were also excluded if they impacted the eye but are not primarily classed as an eye disease (eg, CHARGE (Coloboma of the eye, Heart defects, Atresia of the choanae, Retardation of Growth and development, and Ear abnormalities and deafness syndrome), Behcet’s syndrome). Fuchs endothelial corneal dystrophy (FECD) was included, as the prevalence of this condition is widely variable geographically and thus is listed as a rare disease on Orphanet, ORPHA ID: 98 974.

### Information sources and search terms

Four online databases were searched for identification of articles: MEDLINE, EMBASE, PubMed and Cochrane Database of Systematic Reviews. Grey literature was also searched: OpenGrey and GreyLit. Search terms were generated initially for use in MEDLINE and adapted for the other databases (online supplementary table S1). The population-intervention-comparator-outcome framework was used to develop these search terms: ‘population’ being non-tumorous rare eye diseases, ‘intervention’ being measurement of methylation, no ‘comparator’ was used and ‘outcome’ being implications of differential methylation for rare eye disease research.^38^ As there is no existing registry of rare eye conditions, search terms were curated by referring to the Genetic and Rare Diseases Information Center, the National Eye Institute, the Retina International and the Irish Target 5000—Gateway to Vision. Additionally, rare eye conditions which met ‘The 100,000 Genomes Project’ ophthalmology inclusion criteria were also included.

10.1136/bmjophth-2019-000342.supp1 Supplementary data

### Study selection, data extraction and critical appraisal

Searches and data extraction were last performed in duplicate by two independent personnel on 20 August 2019. Duplicate articles were first removed automatically by reference management software EndNote V.X8. Articles were then screened by publication date, title, abstract and keywords to identify relevant studies and remove any duplicates missed by the reference management software. The remaining articles were screened by reading the full text. Reference lists were screened for reverse citations and a ‘cited reference search’ was performed on Web of Science to identify any potential relevant forward citations. Data extraction was performed, and methodological rigour critically appraised using customised forms based on checklists from the Joanna Briggs Institute critical appraisal tools (online supplementary tables S2 and S3).^39^ Methodological rigour scoring decisions of weak, moderate and strong were based on number of participants, appropriate matching of cases to controls, use of experimental controls and addressing confounding factors. Insufficient homogenous data were available to perform a meta-analysis so a narrative synthesis of the data was undertaken, using the University of Lancaster narrative synthesis guidelines.^40^

## Results

**Figure 1:**
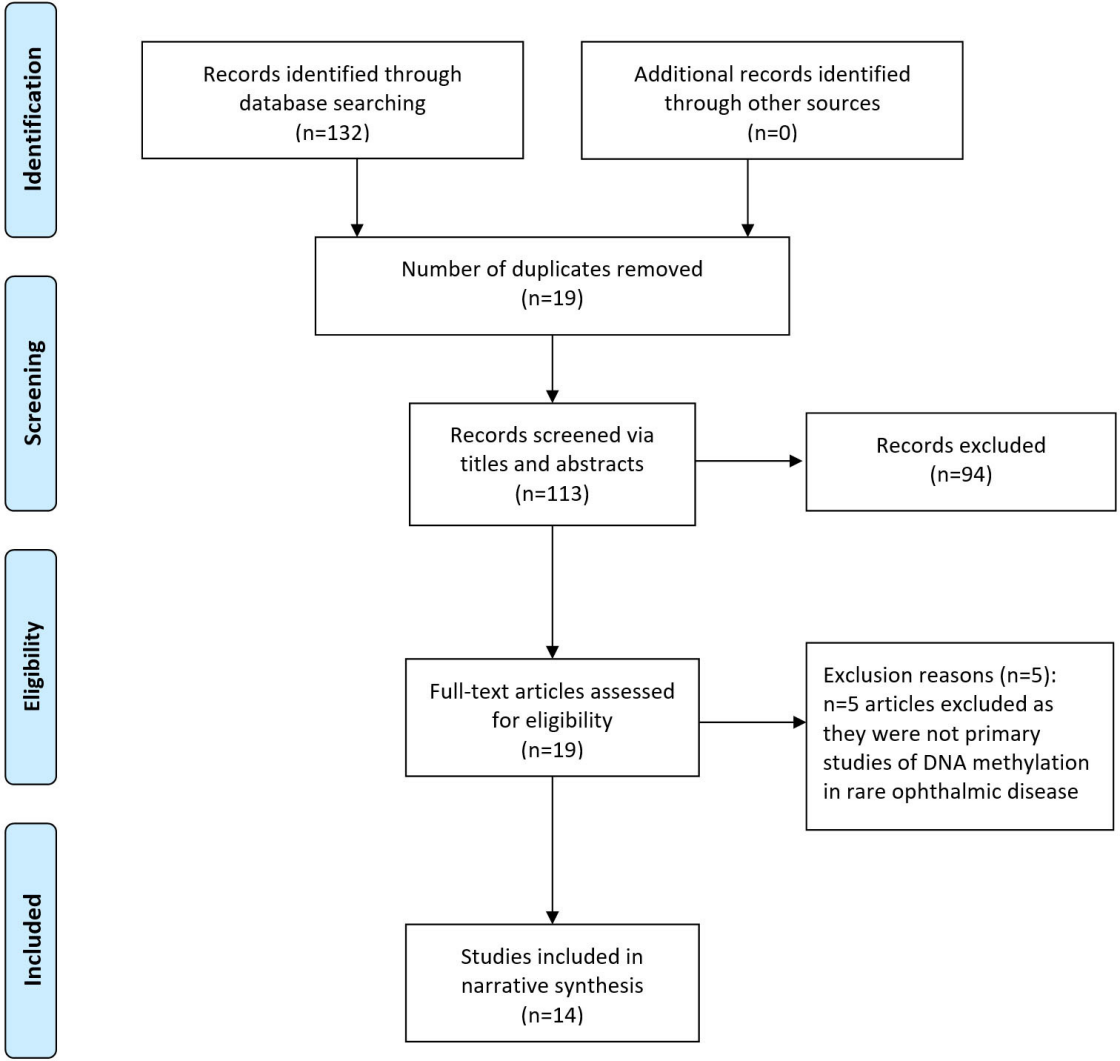
Adapted from the Preferred Reporting Items for Systematic Reviews and Meta-Analyses (PRISMA) flow diagram, summarising screening method and study selection process.

Sources identified per databases were as follows: MEDLINE (n=22), EMBASE (n=75), PubMed (n=32), Cochrane Database of Systematic Reviews (n=3), OpenGrey (n=0) and GreyLit (n=0). No further papers were identified following forward/reverse citation searching. Screening of the 132 sources via title, abstract and keywords resulted in the removal of 19 duplicates and 94 sources were found not to be relevant. This left 19 papers for full-text screening, of which five were found to not be primary studies of methylation in rare ophthalmic disease. Therefore, 14 papers met the inclusion/exclusion criteria for final inclusion in the review. This screening and selection process is summarised in figure 1. Eight of these papers were case–control studies and three were case reports, and three were in vivo interventional studies using murine models. Study characteristics extracted included research aims, methylation measurement, participant information and key results (online supplementary table S4). Methodological rigour was scored as weak for each study based on a variety of factors, including low numbers of participants, a lack of strategies to deal with confounding factors or a lack of appropriately matched experimental controls used. Countries of publication origin included the USA, Japan, Poland, the UK, China, Sweden, India, Germany, Spain, Denmark and South Korea. A marked increased was seen in the number of studies of methylation and rare ophthalmic disease in recent years, with 10/14 (71.4%) studies published within the last decade, of which 40% were published since 2018. A succinct summary of ophthalmic conditions studied, methylation measurement method and key outcomes for these studies is presented in table 1.

**Table 1:** Study characteristics summary of sources included in review

First author, publication year and reference number	Rare eye disease	Methylation measurement method	Participant information	Outcomes
Case–control studies (n=8)				
Ali, 2004^46^	CFEOM1	Bisulfite sequencing of CpG dinucleotides in exon 21 of *KIF21A.*	Ten individuals in an Indian family affected by CFEOM1, with no other clinical abnormalities.	Differential methylation within exon 21 of the *KIF21A* gene contributed to the high mutation rate.
Bulka, 2019^47^	Retinopathy of prematurity	Infinium MethylationEPIC array of bisulfite-treated DNA.	Premature infants with prethreshold retinopathy of prematurity compared with unaffected premature infants.	Five probes were associated with elevated methylation and disease risk. Differential methylation was identified in eight autoinflammatory genes.
Chan, 2018^54^	FECD	Infinium MethylationEPIC array data reanalysed looking at 2227 miRNA probes.	Corneal endothelial tissue from patients with FECD compared with age and sex- matched patients without FECD.	Differential methylation was seen in miRNA genes in FECD, including hypermethylation of *miR-199b-5p* which negatively regulates *Snail* and *ZEB1*.
Jin, 2008^51^	RP	Methylation-specific PCR of bisulfite-treated DNA.	Four patients with RP compared with three unaffected patients.	No differential methylation patterns were observed between healthy controls and RP participants.
Kabza, 2019^50^	Keratoconus	Reduced representation bisulfite sequencing.	Corneal tissue from five patients with keratoconus and five unaffected controls.	18 differentially methylated genes corresponding to expression data were identified in patients with keratoconus.
Khuc, 2017^44^	FECD	HM450 DNA methylation array kit and MethyLight real-time PCR.	Corneal samples from patients with FECD (n=15) undergoing corneal translation surgery and four matched normal corneal endothelium.	59% and 41% of probes displayed hypermethylation and hypomethylation, respectively, in FECD patient samples.
Maeng, 2015^43^	GCD2	ChIP microarray analysis and MeDIA-assisted CpG microarray analysis.	Corneal fibroblasts of four patients with GCD2 compared with normal corneal fibroblast controls (n=3).	Epigenetic modifications by histones affect expression of *TGFBIp* and extracellular matrix genes in corneal fibroblasts.
Porter, 2015^42^	Brittle cornea syndrome	ChIP microarray and mass spectrometry.	Cells from five patients with type 2 brittle cornea syndrome compared with six unaffected controls.	HP1BP3 interactions with H3K9 methylated genomic regions were lost in mutated samples with mutated PRDM5 proteins.
Case report studies (n=3)				
Friedrich, 1993^45^	X linked RP	Methylation analysis of the *XLRP2* gene using a digoxigenin-labelled M27 beta probe.	XLRP2-affected family including seven obligate carrier females and six daughters of obligate carriers with variable clinical phenotypes.	No correlation between differential methylation of X chromosomes and clinical severity in patients with XLRP2.
García‐Hoyos, 2005^48^	Choroideraemia	Methylation-specific PCR of bisulfite DNA.	One female with choroideraemia, with a balanced chromosome X and 4 translocation.	Non-random methylation patterns were seen resulting in skewed X inactivation in the participant with choroideraemia.
Wei, 2015^52^	Congenital cataract	MRSE-PCR was used to measure promoter methylation.	Monozygotic twin sisters, one with congenital pulverulent nuclear cataract and one without.	Differential methylation was not found in candidate genes between siblings.
In vivo interventional studies using murine models (n=3)				
Farinelli, 2014^53^	RP	Immunofluorescence, MeDIP, DNA methylation microarray.	Murine models of RP, known as *rd1* strains, of different strains used irrespective of gender and corresponding wild-type strains.	Binding sites of transcription factors differentially methylated in RP mutant strains. DNMT inhibition reduced photoreceptor cell death.
Montana, 2013^49^	RP	Bisulfite DNA was PCR amplified and CpG methylated detected on bisulfite sequencing DNA methylation analysis software.	Murine models of RP with germ line *Nrl* and *Pde6b* knockouts.	Knockouts of *Nrl* and *Pde6b* resulted in complete reprogramming in germ line mutant mice, and *Nrl/Pde6b* inactivation in adulthood resulted in partial reprogramming of red to cone cell transition, alleviating RP.
Zheng, 2018^41^	RP	Histone methylation measured by TMT labelling and HPLC and mass spectrometry.	Murine models of RP and age-matched wild-type controls.	Treatment with the methyltransferase inhibitor DZNep is followed by reduction in outer nuclear layer cell death.

CFEOM1, congenital fibrosis of extraocular muscles-1; ChIP, chromatin immunoprecipitation; CpG, cytosine-guanine dinucleotide; DNMT, DNA methyltransferase; DZNep, 3-Deazaneplanocin A; FECD, Fuchs endothelial corneal dystrophy; GCD2, granular corneal dystrophy type 2; HM450, Illumina Infinium HumanMethylation450; HPLC, high-performance liquid chromatography; MeDIA, methylated DNA isolation assay; MeDIP, methylated DNA immunoprecipitation; miRNA, microRNA; MRSE, methylation-sensitive restriction enzyme; RP, retinitis pigmentosa; TMT, tandem mass tag labelling; XLRP2, X linked retinitis pigmentosa 2.

## Discussion

### Findings of this systematic review and potentials for future research

Following a comprehensive systematic review of the literature, this study found that the role of methylation in ophthalmic disease has been evaluated in histone methylation marks,^41–43^ gene body and promoter regions,^44–53^ and miRNAs,^54^ in the following rare ophthalmic conditions:

1. Brittle cornea syndrome, an autosomal recessive inherited disorder characterised by corneal fragility.^42^
2. Choroideraemia, an X linked recessive disorder of retinal degeneration.^48^
3. Congenital cataract, a disease characterised by lens opacity at birth.^52^
4. Congenital fibrosis of extraocular muscles-1 (CFEOM1), a disorder characterised by ptsosis and ophthalmoplegia.^46^
5. FECD, a late-onset progressive inherited disorder (typically autosomal dominant inheritance) leading to loss of visual acuity.^44 54^
6. Granular corneal dystrophy type 2 (GCD2), a disease characterised by granular deposits in the cornea leading to progressive vision loss.^43^
7. Keratoconus, a condition characterised by distortion of the corneal surface.^50^
8. Retinitis pigmentosa (RP), a progressive disorder leading to loss of vision through retinal cell degeneration.^41 45 49 51 53^
9. Retinopathy of prematurity, a congenital disorder of retinal cell degeneration resulting from premature birth.^47^

Aberrant methylation was established as a pathogenic biomarker of ophthalmic disease in eight studies. This included: the identification of methylation as playing a role in the high mutability of exon 21 within *KIF21A* in CFEOM1,^46^ loss of H3K9me2-mediated repression in brittle cornea syndrome,^42^ differential methylation of autoinflammatory genes in retinopathy of prematurity (including *NGPT1*, *BDNF*, *CRP*, *MPO*, *SAA1*, *SAA2*, *TNFRSF1A* and *TNFRSF1B*),^47^ differential methylation of several genes corresponding to RNA sequencing gene expression analysis in keratoconus,^50^ hypomethylation in genes related to ion channel roles which are essential for corneal endothelium function in patients with FECD,^44^ differential miRNA expression in FECD cases compared with controls including the hypermethylation of *miR-199b-5p* which negatively regulates the corneal transcription factors *Snail* and *ZEB1*,^54^ altered histone methylation status associated with changes in *TGFBIp* expression levels in GCD2^43^ and, finally, skewed inactivation of the X chromosome associated with a non-random DNA methylation pattern in choroideraemia.^48^

DNA methylation was evaluated as a potential novel therapeutic target in two studies of RP through treatment with methyltransferase inhibitors (decitabine and 3-Deazaneplanocin A) which were found to reduce RP phenotype progression.^41 53^ Methylation inhibitors have been investigated in recent years as therapeutic agents for cancer treatment, due to the role of hypermethylation as an inhibitor of tumour suppressor genes.^55^ Decitabine is a Food and Drug Administration (FDA)-approved drug for treatment of haematological malignancies, and while 3-Deazaneplanocin A is not currently FDA approved, it has been investigated for its role in inhibiting histone methylation in acute myeloid leukaemia and are therefore examples of drug repurposing opportunities for non-cancerous rare ophthalmic disease. Although retinoblastoma and uveal/intraocular melanoma are not included in this review, epigenetics and rare tumorous ophthalmic conditions have been reviewed extensively elsewhere within the last 2 years, with extensive studies illustrating the association of hypermethylation of tumour suppressor gene promoter regions and ophthalmic tumorigenesis.^56 57^ Therefore, the association of methylation with ophthalmic disease, coupled with attenuated retinal degeneration following methyltransferase inhibition in the two cited articles,^41 53^ highlights the need to conduct further investigations into epigenetic inhibitors as therapeutic agents for both tumorous and non-tumorous ophthalmic disease. Additionally, one study identified two candidate genes (*NRL* and *PDE6B*) for targeted gene therapy RP research, with the important caveat that DNA methylation must be considered for reprogramming the transition of adult rod photoreceptor cells to cone cells; adult murine models subjected to *Nrl/Pde6b* inactivation showed only partial reprogramming which was insufficient to alter DNA methylation patterns, and incomplete RP symptom alleviation.^49^

Two studies investigating the potential role of aberrant methylation in congenital cataracts,^52^ and RP,^51^ found no significant difference in methylation levels between cases and controls in the candidate genes selected for analysis. However, in the case report of monozygotic twins discordant for congenital cataracts, the candidate gene *CRYAA* was identified from previous research into common conditions such as age-related cataract; there is a need for whole epigenome analysis to identify differentially methylated genes which may differ between common and rare ophthalmic conditions.^35^ For the second study, methylation analysis was able to aid the exclusion of a candidate gene as having a causative role in RP, where there was a random pattern of methylation and therefore no evidence of imprinting of mutated *FSCN2*.^51^ Furthermore, the case report on a family of individuals affected by X linked RP2 did not find an association between differential methylation in the X chromosome and clinical severity.^45^ However, as this study was conducted in 1993 using an M27 beta probe, it would be interesting to see whether modern comprehensive methods of measuring methylation might show a different result. Additionally, as is true for the majority of epigenetic studies, a longitudinal analysis of epigenetic changes over time would be valuable to determine that differential methylation is a causative factor of disease rather than an incidental change that naturally occurs over a lifetime.

Of the articles included in this review, 71.4% studies were published within the last decade and of these 40% were published since 2018, demonstrating the increasing awareness of the importance of epigenetics in ophthalmic disease and subsequent undertaking of research. However, the rare eye diseases reported in the existing literature are a small subset of existing rare conditions. Difficulties comprising search terms exhaustive of the total list of rare eye conditions reflect the need for a registry of rare eye conditions, as exists for rare renal conditions^58^ and which would be invaluable in future rare ophthalmic disease research. Countries of origin for each publication varied widely from the USA, Japan, Poland, the UK, China, Sweden, India, Germany, Spain, Denmark and South Korea. Building on this, it would be useful to perform comparative methylation analysis on ethnically diverse rare ophthalmic disease populations by encouraging international collaboration.

### Limitations of this systematic review

While a randomised controlled trial (RCT) with many participants is desirable, the scarcity of patients with a given rare disease, coupled with the fact that epigenetic analysis is a developing field, makes ‘gold-standard’ RCTs difficult for researchers to achieve. Therefore, it is unsurprising that one limitation of this review is that the studies included were all scored as having weak methodological rigour based on low participant numbers, lack of addressing confounding factors or insufficient experimental controls described. This review highlights the need for a standardised approach of conducting rare disease and multiomic research, such as the standardised reporting structure that exists for genetic association studies.^59^ Furthermore, several of these studies reported differential methylation in animal models,^41 49 53^ therefore it would be helpful for future research to perform studies of these differentially methylated genes in human biopsies before differential methylation is validated as a clinically useful method of characterising rare ophthalmic disease.

### Clinical relevance of these findings

As is typical of patients with rare conditions, patients with rare ophthalmic conditions can face difficulties obtaining a diagnosis, and treatment for their condition is often limited which can have severely detrimental impacts for a patient’s vision. Therefore, there is a need for improving our understanding of these conditions, to aid diagnosis, provide prognoses or identify therapeutic targets which could correct or halt degeneration in patients with rare eye dysfunction. Projects such as the National Eye Institute’s eyeGene, Target 5000—Gateway to Vision and The 100,000 Genomes Project are important steps towards achieving this goal. Building on this momentum and the advances of next-generation sequencing technology in the last decade, this review highlights how an allied multiomic approach, including genomics and epigenomics, to characterise rare ophthalmic disease is the next step in research. In particular, epigenomics is an encouraging place to start, having been reported as associated with in common eye conditions such as diabetic retinopathy, age-related macular degeneration and acquired cataracts.^60^

## Conclusions

Differential methylation has been identified in a small number of rare ophthalmic disease studies, emphasising the need for further research projects like eyeGENE, Target 5000—Gateway to Vision and The 100,000 Genomes Project. Yet the limited evidence that is available is promising, and emphasises the potential for molecular characterisation of rare eye disorders using methylation. Highlights include:

- Identification of increased methylation in the outer nuclear layers and reduced photoreceptor cell death following treatment with a DNA methyltransferase inhibitors decitabine and 3-Deazaneplanocin A in RP models.
- Reprogramming of adult rod cells through *Nrl* inactivation as a novel therapeutic target of RP in murine models.
- Differential methylation of genes identified related to corneal endothelium function in FECD independent of age or gender.
- Identification of epigenetic histone modifications as having a disease-causing role in GCD2.

These projects provide a platform for future multiomic approaches to study rare ophthalmic disease and the much needed development of a standardised analysis and reporting approach. Integrating epigenomic markers such as DNA methylation with genomic analysis has the potential to aid diagnosis, limit loss of patient vision and ultimately improve patient independence and quality of life.
