# Supplementary material for: Systematic review of differential methylation in rare ophthalmic diseases
Source: BMJ Open Ophthalmol. 2019 Nov 13;4(1):e000342. doi: 10.1136/bmjophth-2019-000342 (PMC6861117; doi:10.1136/bmjophth-2019-000342)
Supplement: Supplementary data [file bmjophth-2019-000342supp001.pdf]

Supplementary materials and methods

A systematic review of differential methylation in rare ophthalmic diseases.

Ms Katie Kerr, Dr Helen McAneney, Dr Laura Smyth, Dr Cheryl Flanagan, Ms Giuliana Silvestri, Dr Andrew Nesbitt, Mr Christopher Wooster, Dr Amy Jayne McKnight.

Table S1. Search terms used in MEDLINE and adapted for other databases.

#	Search term
1.	(Achromatopsia or ACHM or Pingelapese blindness or Rod monochromacy or Rod monochromatism).mp.
2.	(Acute zonal occult outer retinopathy or AZOOR).mp.
3.	(Adult-onset vitelliform macular dystrophy or Adult-onset foveomacular dystrophy or Gass disease or Pseudo-Best disease or Pseudo-vitelliform macular dystrophy).mp.
4.	(Aland islands eye disease or Forsius-Eriksson syndrome or Forsius-Eriksson type ocular albinism).mp.
5.	(Anterior uveitis or Iridocyclitis).mp.
6.	(Autosomal dominant keratitis or Hereditary Keratitis).mp.
7.	(Autosomal recessive isolated optic atrophy or Autosomal recessive nonsyndromic optic atrophy).mp.
8.	(Autosomal recessive primary microcephaly or Microcephalia vera).mp.
9.	(Axenfeld-Rieger syndrome or Axenfeld syndrome or Rieger syndrome).mp.
10.	(Best Disease or Best vitelliform macular dystrophy or Early-onset vitelliform macular dystrophy or Juvenile-onset vitelliform macular dystrophy or Polymorphic vitelline macular degeneration or Vitelliform macular dystrophy type 2).mp.
11.	(Biettis Crystalline Dystrophy or Bietti crystalline corneoretinal dystrophy).mp.
12.	(Bilateral corneal abnormality or bilateral corneal abnormalities).mp.
13.	(Birdshot chorioretinopathy or Birdshot chorioretinitis or Birdshot retinochoroiditis or Birdshot retinochoroidopathy or Vitiliginous choroiditis).mp.
14.	(Bradyopsia or Prolonged electroretinal response suppression).mp.
15.	(Central areolar choroidal dystrophy or Central areolar choroidal sclerosis).mp.
16.	(Choroideremia or Tapetochoroidal dystrophy).mp.
17.	(Coats disease or Congenital retinal telangiectasia or Leber miliary aneurysm).mp.
18.	(Congenital cataract or Cataract Hutterite type or Early-onset partial cataract or Total early-onset cataract or Cerulean cataract or Blue-dot cataract or Cataract microcornea syndrome).mp.
19.	(Congenital foveal retinoschisis or foveal hypoplasia).mp.
20.	(Congenital glaucoma or Juvenile glaucoma or Hereditary glaucoma or Primary glaucoma).mp.
21.	(Congenital macula abnormality or North Carolina Macular dystrophy).mp.
22.	(Congenital microcoria or Congenital miosis).mp.
23.	(Doyne honeycomb retinal dystrophy or Familial drusen).mp.
24.	(Duane syndrome or Stilling-Turk-Duane syndrome or Duane retraction syndrome).mp.

25. (Eales disease or Idiopathic retinal perivasculitis or Idiopathic retinal vasculitis).mp.
26. (Fish-eye disease or Partial LCAT deficiency).mp.
27. (Goldmann-Favre syndrome or Enhanced S-cone syndrome).mp.
28. (Gyrate atrophy or Hyperornithinemia or Ornithine aminotransferase deficiency).mp.
29. (Idiopathic retinal vasculitis-aneurysms-neuroretinitis syndrome or IRVAN syndrome).mp.
30. (Isolated anophthalmia-microphthalmia syndrome or Microphthalmia-anophthalmia-coloboma spectrum or MAC spectrum or Colobomatous microphthalmia or Nanophthalmia).mp.
31. (Isolated congenital megalocornea or Congenital anterior megalophthalmia).mp.
32. (Isolated optic neuritis or Chronic relapsing inflammatory optic neuropathy).mp.
33. (Leber Congenital Amaurosis or Early-Onset Severe Retinal Dystrophy).mp.
34. (Microcornea-rod-cone dystrophy-cataract-posterior staphyloma syndrome or MRCS syndrome).mp.
35. (Norrie disease or Atrophia bulborum hereditaria or Episkopi blindness or Norrie-Warburg disease).mp.
36. (Oguchi disease or Oguchi syndrome or Congenital stationary night blindness or Oguchi type or Rod dysfunction syndrome or fundus albipunctatus).mp.
37. (Oligocone trichromacy or Oligocone syndrome).mp.
38. (Stern-Lubinsky-Durrie syndrome or Corneodermatoosseous syndrome).mp.
39. (Superior limbic keratoconjunctivitis or Theodore's superior limbic keratoconjunctivitis).mp.
40. (Tolosa Hunt syndrome or Painful ophthalmoplegia).mp.
41. (Usher Syndromes or Retinitis pigmentosa-deafness syndrome).mp.
42. (Vernal keratoconjunctivitis or Spring catarrh).mp.
43. “Retinopathy of prematurity”/
44. Albinism, Ocular/
45. ANIRIDIA/
46. Autosomal dominant optic atrophy.mp.
47. Borholm eye disease.mp.
48. Brittle cornea syndrome.mp.
49. Chandler's syndrome.mp.
50. COLOBOMA/
51. Cone Rod Dystrophies/
52. Congenital ectropion.mp.
53. Congenital extraocular muscle fibrosis.mp.
54. Congenital primary aphakia.mp.
55. Corneal Dystrophies, Hereditary/
56. Familial conjunctiva pterygium .mp.
57. Familial exudative vitreoretinopathy.mp.
58. Fuchs' Endothelial Dystrophy/
59. Fuchs heterochromic iridocyclitis.mp.
60. Hereditary vascular retinopathy.mp.
61. Infantile nystagmus.mp.
62. Inherited macular dystrophy.mp.
63. Iridocorneal endothelial syndrome.mp.

64. Isolated ectopia lentis.mp.
65. Juvenile Macular Degeneration.mp.
66. Kearns-Sayre Syndrome/
67. Keratoconus/
68. Late-onset retinal degeneration.mp.
69. Lenz microphthalmia syndrome.mp.
70. Morning glory syndrome.mp.
71. Oculoauriculofrontonasal syndrome.mp.
72. Oculocerebral dysplasia.mp.
73. Oculomotor apraxia Cogan type.mp.
74. Optic perineuritis.mp.
75. PANUVEITIS/
76. Patterned dystrophy retinal pigment epithelium.mp.
77. Peters anomaly.mp.
78. Progressive bifocal chorioretinal atrophy.mp.
79. Retinitis Pigmentosa/
80. Ring dermoid of cornea.mp.
81. Snowflake vitreoretinal degeneration.mp.
82. Stargardt Disease.mp.
83. Syndromic microphthalmia.mp.
84. Visual snow syndrome.mp.
85. Vitreoretinochoroidopathy dominant.mp.
86. X-linked retinoschisis.mp.
87. (rare eye disease* or rare eye disorder* or rare ophthalmic disease* or rare ophthalmic disorder*).mp.
88. (methylation or methylated or hypermethylated or hypomethylated or hypermethylation or hypomethylation).mp.
89. 1 or 2 or 3 or 4 or 5 or 6 or 7 or 8 or 9 or 10 or 11 or 12 or 13 or 14 or 15 or 16 or 17 or 18 or 19 or 20 or 21 or 22 or 23 or 24 or 25 or 26 or 27 or 28 or 29 or 30 or 31 or 32 or 33 or 34 or 35 or 36 or 37 or 38 or 39 or 40 or 41 or 42 or 43 or 44 or 45 or 46 or 47 or 48 or 49 or 50 or 51 or 52 or 53 or 54 or 55 or 56 or 57 or 58 or 59 or 60 or 61 or 62 or 63 or 64 or 65 or 66 or 67 or 68 or 69 or 70 or 71 or 72 or 73 or 74 or 75 or 76 or 77 or 78 or 79 or 80 or 81 or 82 or 83 or 84 or 85 or 86 or 87
90. 88 and 89

Table S2. Template quality appraisal form for case control studies.

		Yes	No	Unclear	Not Applicable	Comments
1	Was the primary focus of the paper methylation?					
2	Were the groups comparable other than the presence of disease in cases or the absence of disease in controls?					
3	Were cases and controls matched appropriately?					
4	Was methylation measured in in a standard reliable way?					
5	Was methylation measured in the same way for cases and controls?					
6	Were confounding factors identified?					
7	Were strategies to deal with confounding factors identified?					
8	Was the exposure period of interest long enough to be meaningful?					
9	What statistical analysis was used and was this analysis appropriate?					
10	Were experimental controls used?					

Table S3. Template quality appraisal form for case report studies.

	Yes	No	Unclear	Not Applicable	Comments

1	Was the primary focus of the paper methylation?					
2	Was the patient’s history described clearly?					
3	Was the patient's current clinical condition described clearly?					
4	Was methylation measured in in a standard reliable way?					
5	Were confounding factors identified?					
6	Were strategies to deal with confounding factors identified?					
7	Were experimental controls used?					

Table S4. Study characteristics data extraction form.

First author, and publication year	Title and digital object identifier	Research objective	Design information	Key results
Ali, M.<br>2004<br><br>Reference number: 46	Mutation analysis of the *KIF21A* gene in an Indian family with CFEOM1[a]: implication of CpG[b] methylation for most frequent mutations<br><br>DOI:<br>10.1080/13816810490498198	To identify the molecular explanation for the most frequent mutations in a trans-generational Indian family: c.2860C>T and c.2861G>A in exon 21 of the *KIF21A* gene.	Observational design: case control<br><br>Population:<br>Ten individuals from a four-generation Indian family with CFEOM1 (unaffected family members used as comparisons) and no other abnormalities.<br><br>Methylation measurement: Bisulphite sequencing of blood and sperm genomic DNA[c] to analyse CpG dinucleotides in exon 21 of the *KIF21A* gene, commonly mutated in CFEOM1.<br><br>Methodological rigour: weak	Mutational analysis of a PCR[d] product from an affected individual identified the mutation c.2860C>T and, although less commonly, c.2861G>A in exon 21 of the *KIF21A* gene. These mutations combined accounted for 84% of the CFEOM1 cases. As both these mutations occurred in a CpG dinucleotide, methylation status was analysed by bisulphite sequencing.<br><br>Bisulphite sequencing found that the c.2860 and 2861 sites were methylated and that this methylation in part may account for the high mutability of these positions.
Bulka, C.M.<br>2019<br><br>Reference number: 47	Placental CpG Methylation of Inflammation, Angiogenic, and Neurotrophic Genes and Retinopathy of Prematurity.<br><br>DOI:<br>10.1167/iovs.18-26466	To investigate the role of CpG methylation in preterm infants risk of retinopathy of prematurity.	Observational design: case-control.<br><br>Population:<br>Pre-term newborn babys with prethreshold retinopathy of prematurity at 25.1 gestational weeks compared to newborn babies who did not at 26.1 gestational weeks.<br><br>Methylation measurement:<br>Illumina Infinium MethylationEPIC array used to analyse bisulphite converted DNA samples.<br><br>Methodological rigour: weak	Increased methylation levels at eleven probes was associated with a decreased risk of retinopathy of prematurity (probes: (cg10266336, cg15484375, cg12907644, cg06979684, cg00473501, cg01565803, cg07694252, cg06740893, cg11151395, cg14619064, and cg22331200), while increased methylation in five different probes was associated with an increased risk of retinopathy of prematurity (cg14970975, cg05199346, cg07159484, cg15483907, and cg00989220).<br>Most significantly differentially methylated probes were within the autoinflammatory genes:<br>*ANGPT1*, *BDNF*, *CRP*, *MPO*, *SAA1*, *SAA2*, *T NFRSF1A*, and *TNFRSF1B.*
Chan, M.F.<br>2018	Aberrant DNA methylation of	Targeted and genome wide	Observational design: case-control.	Hierarchical clustering displayed differential miRNA methylation between cases and

Reference number: 54	miRNAs in Fuchs endothelial corneal dystrophy<br><br>DOI: 10.1101/638486.	characterisation of miRNA[e] methylation changes in patients with FECD[f].	Population:<br>Corneal endothelium samples were collected from FECD patients alongside corneal endothelium samples from age and sex matched non-FECD patients.<br><br>Methylation measurement:<br>Methylation array data collected in a previous study, [44] was subjected to subanalysis focusing on 2,227 miRNA probes of 463 miRNA genes. MethyLight analysis was also performed on an additional cohort of FECD cases and controls.<br><br>Methodological rigour: weak	controls, predominantly DNA hypermethylation (71%, 154 probes, of FECD samples were hypermethylated compared to just 29%, or 62 hypomethylated probes). Differential methylation was predominantly seen within promoter regions (96%) and no significant methylation changes were seen with reference to age or sex.<br><br>Of the 154 hypermethylated probes, 74% were within intragenic sequences. Similarly, 71% of the 62 hypomethylated probes occurred in intragenic sequences, 44% of these being within intronic regions.<br>MethyLight validation confirmed the differential methylation between cases and controls from the HumanMethylation450 array, with higher mean DNA methylation in FECD patients, as well as specific methylation of the miRNAs: *miR-199A1* and *miR-23B* in FECD cases compared to controls.<br><br>An inverse relationship between hypermethylation and gene expression levels was see in 18 miRNAs, in particular the almost complete silencing of *miR-199b-5p* expression (which contained the highest level of promoter hypermethylation), suggesting a role in FECD pathogenesis.<br><br>A dual-luciferase reporter assay found that *miR-199b-5p* directly regulated Snail and ZEB1 mRNA transcripts, reducing expression levels significantly in human corneal endothelial cells.
Farinelli, P.<br>2014<br><br>Reference number: 53	DNA methylation and differential gene regulation in	To elucidate patterns of differential DNA methylation	*In vivo* interventional study using murine models.<br>Population:	In all RP mice models, there was a general increase of methylation in the outer nuclear layer compared to any retinal layer of healthy

	photoreceptor cell death<br><br>DOI: 10.1038/cddis.2014.512	between RP[g] tissue and healthy tissue and identify if DNA methylation is a potential new therapeutic target.	Mice and rat models of RP, known as *rd1* strains, used irrespective of gender and corresponding wild type strains. Ages were used corresponding to stages of retinal degeneration.<br><br>Methylation measurement: Immunofluorescence using anti-5 methylcytosine antibody, MeDIP[h], DNA methylation microarray and gene mapping using 'cistrome platform peak2gene' software.<br><br>Methodological rigour: weak	wild types, but not in the inner retinal layers of RP models.<br><br>5methylcystosine antibody co-labelled with dying cells in terminal deoxynucleotidyl transferase dUTP nick end labelling assay, indicating a link between DNA methylation and photoreceptor degeneration, particularly evident at the late stages of photoreceptor cell death.<br><br>DNA methylation measurement at whole tissue level found little difference between *rd1* and *wt*, indicating that in contrast to cellular approach, whole tissue measurement of methylation may be insufficiently sensitive to detect a small number of cells showing strong DNA methylation.<br><br>Microarray analysis showed 1284 hypermethylation genes in *rd1* compared to *wt* in intragenic regions.<br><br>Hypermethylation of *rd1* mice appeared to be greater in binding motifs of transcription factors YY1, E2F3 and NRL, all of which have possible roles in RP, and their target genes compared to *wt*.<br><br>DNMT[i] inhibition using decitabine increased survival of photoreceptors in short term cultures (4 days) from *rd1* animal retinae compared to control (not treated) but not in long term cultures (11 days).
Freidrich, U.<br>1993<br><br>Reference number: 45	X-inactivation pattern in carriers of X-linked retinitis pigmentosa: A valuable means of	Investigation of methylation patterns in a family with XLRP2[j] to determine if a	Observational design: case report<br><br>Population:<br>A family with XLRP2, including seven obligate carrier females and six daughters	In patients with XLRP, the abnormal X chromosome appears to be active in nearly all cells.

	prognostic evaluation?<br><br>DOI: 10.1007/BF01247335	correlation exists between clinical severity and preferential X chromosome inactivation.	of obligate carriers. Clinical phenotype ranged from partial vision impairment to complete vision loss.<br><br>Methylation measurement: Conducted using the digoxigenin-labeled M27 beta probe to examine the *XLRP2* gene methylation.<br><br>Methodological rigour: weak	The opposite methylation pattern was observed in a phenotypically normal carrier.<br><br>There is a potential association between extreme skewed X chromosome inactivation and clinical severity.<br><br>No correlation between differential methylation status of X chromosomes and clinical severity.
García-Hoyos, M.<br>2005<br><br>Reference number: 48	New approach for the refinement of the location of the X-chromosome breakpoint in a previously described female patient with choroideremia carrying a X;4 translocation.<br><br>DOI: 10.1002/ajmg.a.30987	Elucidation of the role of the X chromosome breakpoints in choroideremia pathogensis.	Observational design: case report.<br><br>Population:<br>Female individual with choroideremia previously found to possess a balanced translocation between chromosomes X and 4.<br><br>Methylation measurement:<br>Methylation specific PCR analysis of bisulphite treated DNA.<br><br>Methodological rigour: weak	Expression analysis revealed the chromosomal breakpoint between primers CHM-4 and CHM-C. Skewed inactivation (almost complete inactivation) of the X chromosome was seen by the methylation specific PCR assay to be in a non-random pattern.
Jin, Z.B.<br>2008<br><br>Reference number: 51	Allelic copy number variation in FSCN2 detected using allele-specific genotyping and multiplex real-time PCRs.<br><br>DOI: 10.1167/iovs.07-1656	Investigation of the *FSCN2* c.72delG mutation in patients with RP.	Observational design: case-control.<br><br>Population:<br>Three RP and three normal control participants, as well as a fourth additional RP participant with an asymmetric allele ratio of 4:1 including an extra copy of the wild-type allele.<br>Methylation measurement:<br>Bisulphite conversion of DNA was processed using a methylation specific PCR assay, followed by cloning of PCR products in a vector (pCR4-TOPO) which	Bisulphite converted DNA from 20 clones showed no significantly different methylation pattern between healthy controls and RP participants, as well as no significant difference in methylation levels between alleles containing the *FSCN2* c.72delG mutation and alleles without, indicating no preferential monoallelic imprinting.

			were DNA sequenced and analysed using the CpGviewer software.<br>Methodological rigour: weak	
Kabza, M.<br>2019<br>Reference number: 50	Multiple Differentially Methylated Regions Specific to Keratoconus Explain Known Keratoconus Linkage Loci.<br><br>DOI: 10.1167/iovs.18-25916	Investigation of the role of DNA methylation in the pathogenesis of keratoconus.	Observational design: case-control<br><br>Population:<br>Five human corneal samples from participants with keratoconus and five from participants without keratoconus.<br><br>Methylation measurement:<br>Reduced representation bisulphite sequencing on the Illumina HiSeq 1500 platform.<br><br>Methodological rigour: weak	112 differentially methylated regions were detected in keratoconus samples, overlapping several loci previously identified as altered in keratoconus<br>(3p14.3, 5q35.2, 13q32.3, 15q24.1, and 20p13.) Additionally differentially methylated regions were detected on the following chromosome arms which have also been previously linked to the keratoconus phenotype: 2q, 4q, 5p, 9p, 14q, and 17q.<br><br>Comparison to RNA sequencing datasets revealed 18 overlapping genes between expression and methylation analysis, including: *IQGAP2*, *SYNJ2*, *CYP1B1*, *MYO1G*, *WNT5A*, *PARVB*, *MGLL*, *CDC25B*, *PSG3*, *FHL2*, *CAMK 1D*, and *THEMIS* (all with decreased expression) and<br>*WNT3*, *RB1*, *AC098617.1*, *RPS6KA2*, *PELI2*, and *PLXNA4* (all with increased expression levels)
Khuc, E.<br>2017<br><br>Reference number: 44	Comprehensive characterization of DNA methylation changes in Fuchs endothelial corneal dystrophy<br><br>DOI: 10.1371/journal.pone.0175112	To compare the DNA methylation landscape of FECD samples to normal samples and elucidate any potential role of methylation in the development of FECD.	Observational design: case control<br><br>Population:<br>Patients with FECD (n=15) undergoing corneal translation surgery (only nine yielded sufficient DNA for analysis) and four age/sex matched normal corneal endothelium.<br><br>Methylation measurement:<br>HM450^{k} DNA methylation array kit and validation performed using MethyLight real time PCR.<br><br>Methodological rigour: weak	Of the probes for *COL8A1, TCF4, SLC4A11,* and *AGBL* genes, previously identified to be associated with late onset FECD, the vast majority did not yield a statistically significant difference in methylation with the exception of *SLC4A11* (14/16 probes). However, 10,961 other probes showed a significant difference in methylation with a false discovery rate of 0.01. Of these, 59% displayed hypermethylation and 41% displayed hypomethylation. No probes were significantly differentially methylated when comparing age or sex.<br><br>Gene ontology analysis showed gene body hypomethylation occurred disproportionately in

				genes of FECD patients related to fluid and ion channel roles, vital for corneal endothelium function as well as cytoskeletal organisation, whilst DNA hypermethylation occurred disproportionately in hematopoietic differentiation in immune system processes.
Maeng, Y. S.<br>2015<br><br>Reference number: 43	Histone methylation levels correlate with *TGFβ1* and extracellular matrix gene expression in normal and granular corneal dystrophy type 2 corneal fibroblasts<br><br>DOI:<br>10.1186/s12920-015-0151-8	Investigation of the role of H3Kme3[l] in *TGFβ1* contributing to GCD2[m].	Observational design: case control<br><br>Population:<br>Corneal fibroblasts of patients with heterozygous GCD2 (n=1) and homozygous GCD2 (n=3) compared to normal corneal fibroblast controls (n=3).<br><br>Methylation measurement:<br>ChIP[n] microarray analysis and MeDIA[o]-assisted CpG microarray analysis.<br><br>Methodological rigour: weak	Levels of H3K4me3 were high in wild type cells compared to GCD2 cells in *TGFβ1* promoter region, whilst H3K27me3[p] levels were low in both cells. DNA methylation levels were not significantly different between the two cell types.<br><br>*TGFβ1* increased active H3K4me1[q] and H3K4me3 levels in *TGFβ1* and other extra cellular matrix associated gene promoters in *wt* fibroblasts but not in GCD2-homozygous cells, correlated with increased expression of *TGFβ1* induced genes. The repressive H3K27me3 decreased in wildtype fibroblasts following *TGFβ1* treatment, but again no effect was found in GCD2-homozygous cells.<br><br>Increases in H3K4me1/3 and decreases in H3K27me3 at *TGFBIp* and extra cellular matrix gene promoters plays a role in GCD2 pathogenesis.

Montana, C.L.<br>2013<br><br>Reference number: 49	Reprogramming of adult rod photoreceptors prevents retinal degeneration.<br><br>DOI: 10.1073/pnas.1214387110	To investigate if developmental reprogramming of adult rods into cone cells could prevent cone loss and the RP phenotype.	*In vivo* interventional study using murine models.<br><br>Population:<br>Murine models with germ-line *Nrl* and *Pde6b* knock outs.<br><br>Methylation measurement:<br>Bisulphite converted DNA from murine retinal tissue was amplified via PCR and analysed using the bisulphite sequencing DNA methylation software to retrieve CpG methylation quantification.<br>Methodological rigour: weak	Germline deletion of *Nrl* on *Pde6b* retinal degeneration mice, as well as condition in activation of the *Nrl* gene in adult mice, supresses the retinal degeneration phenotype through rod gene degeneration and gain of cones. However, only partial re-programming was seen at P44 in *Nrl* knock out mice compared to embryonic *Nrl* knock out as this was insufficient to reprogramme DNA methylation patterns (unchanged at the *Rho* and *Opn1sw* loci), suggesting DNA methylation of these loci may play a role in RP development.
Porter, L.F.<br>2015<br><br>Reference number: 42	A role for repressive complexes and H3K9 di-methylation in PRDM5-associated brittle cornea syndrome.<br><br>DOI: 10.1093/hmg/ddv345	Investigation of the role of *PRDM5* in type 2 brittle cornea syndrome pathogenesis through its epigenetic effects.	Observational design: case control.<br><br>Population:<br>Samples from five patients with type 2 brittle cornea syndrome (n=4 skin and eye fibroblasts, n=1 expression construct), compared to six samples from controls eye pathology (n=4 skin fibroblasts, n=1 post-mortum eye sample and n=1 post-enucleation from corneal trauma sample).<br><br>Methylation measurement:<br>ChIP microarray and mass spectrometry was used to measure PRDM5 and its interaction with histone methyltransferases.<br><br>Methodological rigour: weak	PRDM5 proteins were found to interact with HP1BP3, which binds to H3K9 methylated genomic regions. This interaction was found to be lost in mutated PRDM5 proteins. H3K9me2 was enriched in unaffected controls at the genes *COL13A1, NTN1* and *COL15A1*, and decreased in patients with type 2 brittle cornea syndrome compared to unaffected controls. Therefore, H3K9me2 mediated repression is thought to have a role in normal vascular development and maintenance through PRDM5-target genes.
Wei, T.<br>2015	Exome Sequencing and	To investigate how mutations in coding	Observational design: case report.	Whole exome sequencing analysis followed by Sanger sequencing validation failed to find

Reference number: 52	Epigenetic Analysis of Twins Who Are Discordant for Congenital Cataract.<br><br>DOI: 10.1017/thg.2015.34	regions of candidate genes, as well as differential promoter methylation, may contribute to discordant cataract in monozygotic twins.	Population:<br>Monozygotic twin sisters, one with congenital pulverulent nuclear cataract and one without. Diagnosis was made at age seven and blood samples withdrawn at the time from both sisters.<br><br>Methylation measurement:<br>MRSE-PCR[r] was used to measure promoter methylation.<br><br>Methodological rigour: weak	differential mutations in any of 10 candidate genes *(PLEKHO2, FRYL, RBP3, P2RX2, GSR, TRAM1, VEGFA, NARS2, CADPS* and *TEKT4).*<br><br>Hypermethylation occurred on the promoter region of all six candidate genes analysed for differential methylation (*TRAM1, CRYAA, HSF4, VEGFA, GJA3,* and *FTL*) in both twins. Therefore, differential methylation was not identified to contribute to bilateral congenital cataract in the affected twin.
Zheng, S.<br>2018<br><br>Reference number: 41	DZNep inhibits H3K27me3 deposition and delays retinal degeneration in the rd1 mice.<br><br>DOI:10.1038/s41419-018-0349-8	To investigate the role of histone methylation in RP and potential for histone methylation inhibition as a novel therapeutic target for treatment of RP.	*In vivo* interventional study using murine models.<br><br>Population:<br>Murine models of RP, known as rd1 mice and age matched wild-type controls.<br><br>Methylation measurement:<br>Histone methylation measured by TMT[s] labelling and lysine trimethylation affinity enrichment followed HPLC[t] and mass spectrometry. Results validated using the EpiQuik H3K27me3 quantification kit.<br><br>Methodological rigour: weak	At post-natal day 10 (P10) there is consistently higher histone 3 lysine trimethylation in *rd1* mice compared to *wt* controls. The trimethyl lysine antibody pantrimethyllysine detected 20-30% increase between *rd1* and *wt* controls.<br><br>At P14, *rd1* mice treated with the methyl transferase inhibitor 3-deazaneplanocin A displayed significant reduction in death of outer nuclear layer cells compared to those treated with a phosphate buffer saline control, confirmed to be rod cells (231.5 ± 61.6 cells/mm^2 to 133 ± 35.2 cells/mm^2). No significant reduction at P21.<br><br>Calpain, a protease previously shown to be involved in photoreceptor decay, was decreased following treatment with 3-deazaneplanocin A as well as H3K27me3. However, no significant reduction in H3K9me3[u] or H3K79me3[v] was found.<br><br>RNA[w] sequencing of 3-deazaneplanocin A and phosphate buffer saline treated *rd1* and *wt* respectively showed significantly altered transcriptomes through analysis of differentially

				expressed genes. Interestingly H3K27me3 was found to be the most common histone modification of *rd1* related differentially expressed genes.

Abbreviations.

[a]CFEOM1: congenital fibrosis of extraocular muscles-1, [b]CpG: 5'-C-phosphate, [c]DNA: Deoxyribonucleic acid, [d]PCR: polymerase chain reaction, [e]miRNA: microRNA, [f]FECD: Fuch's endothelial corneal dystrophy, [g]RP: retinitis pigmentosa, [h]MeDIP: methylated DNA immunoprecipitation, [i]DNMT: DNA methyl transferase, [j]XLRP2: X-linked Retinitis Pigmentosa 2, [k]HM450: Illumina Infinium HumanMethylation450, [l]H3Kme3: histone 3 Lysine trimethylation, [m]GCD2: granular corneal dystrophy type 2, [n]ChIP: chromatin immunoprecipitation, [o]MeDIA: methylated DNA isolation assay, [p]H3K27me3: histone 3 lyisne 27 trimethylation, [q]H3K4me1: histone 3 lysine 4 methylated, [r]MRSE-PCR: methylation sensitive restriction enzyme-polymerase chain reaction, [s]TMT: tandem mass tag labelling, [t]HPLC: high performance liquid chromatography, [u]H3K9me3: Histone 3 Lysine 9 trimethylation, [v]H3K79me3: histone 3 lysine 79 trimethylation, [w]RNA: ribonucleic acid.
